# Amassing of heavy metals in soils, vegetables and crop plants irrigated with wastewater: Health risk assessment of heavy metals in Dera Ghazi Khan, Punjab, Pakistan

**DOI:** 10.3389/fpls.2022.1080635

**Published:** 2023-01-16

**Authors:** Muhammad Imran Atta, Syeda Sadaf Zehra, Dong-Qin Dai, Habib Ali, Khalid Naveed, Iftikhar Ali, Muhammad Sarwar, Basharat Ali, Rashid Iqbal, Sami Bawazeer, Usama K. Abdel-Hameed, Iftikhar Ali

**Affiliations:** ^1^ Department of Botany, The Islamia University of Bahawalpur, Bahawalpur, Pakistan; ^2^ Center for Yunnan Plateau Biological Resources Protection and Utilization, Yunnan Engineering Research Center of Fruit Wine, College of Biological Resource and Food Engineering, Qujing Normal University, Qujing, China; ^3^ Department of Agricultural Engineering, Khwaja Fareed University of Engineering and Information Technology, Rahim Yar Khan, Pakistan; ^4^ Department of Plant Pathology, Sub campus Depalpur, University of Agriculture, Faisalabad, Pakistan; ^5^ Department of Agronomy, University of Agriculture, Faisalabad, Pakistan; ^6^ Department of Agronomy, Faculty of Agriculture and Environment, The Islamia University of Bahawalpur, Bahawalpur, Pakistan; ^7^ Umm Al-Qura University, Faculty of Pharmacy, Department of Pharmacognosy, Makkah, Saudi Arabia; ^8^ Biology Department, College of Science, Taibah University, Al-Madinah Al-Munawarah, Saudi Arabia; ^9^ Botany Department, Faculty of Science, Ain Shams University, Cairo, Egypt; ^10^ Center for Plant Sciences and Biodiversity, University of Swat, Charbagh, Pakistan; ^11^ Department of Genetics and Development, Columbia University Irving Medical Center, New York, NY, United States

**Keywords:** heavy metals, wastewater, vegetables, daily metal intake, health risk assesment

## Abstract

Human health is the main concern related to use of crop products irrigated with contaminated irrigation sources. Present research has been conducted to explore heavy metal status of sewage and industrial wastewater being used up for irrigation purpose in the peri-urban areas of the district Dera Ghazi Khan which has not been explored widely before. The analysis also followed heavy metal detection in the subsequent irrigated soil and vegetables/crop plants in relation to assessment of health risk to the consumer to plan the future monitoring in this area. An unremitting boost of heavy metals into the environment from wastewater irrigation has become a global issue. These heavy metals enter the food chain and pose health assumptions to consumers upon utilization. In the present study, an investigation has been conducted to determine metal concentrations in the wastewater, soil, and different plant species. For wastewater samples, pH, total dissolved solids (TDS), electrical conductivity (EC), and selected heavy metals such as Al, As, Cr, Cu, Fe, Mn, Pb, Zn, and Ni were determined. The mean values of heavy metals in the soil samples were within the WHO/FAO safe limit, while Cr and Pb were the most frequent (100%) among the metals. However, differentiating the sites, the concentration of Cr and Cu, Ni, and Fe were elevated. The metal transfer was highly effective from soil to the growing plants i.e. brinjal, red corn, wheat, tomato, and spinach than other plant species. Among the metals, Cr, Ni, Mn, and Pb in plant samples were exceeding the WHO/FAO safe limit. Health risk index (HRI) have revealed the possible potential risk of heavy metal contaminated plant species in the order of spinach (6.4) > wheat (6.4) > brinjal (5.9) > tomato (4.7) > red corn (4.5) > apple gourd (4.3) > white corn (3.8) > cabbage (3.1) > luffa (2.9). Likewise, HRI of different metals was calculated as Cu (19.6) > Zn (17.9) > Cr (2.95) > Ni (0.85) > Mn (0.48) > Fe (0.15) > Cd (0.11) > Pb (0.05) > As (0.00001). The level of HRI through the use of dietary plants revealed an elevated risk level than the acceptable limit (HRI > 1) for Cu > Zn > Cr in adults. Our findings suggest that there would be a serious health risk to the consumers due to the consumption of these plant species being irrigated with the wastewater. Therefore, a strict regulatory mechanism is proposed for the safety of food plants in the study area including monitoring and recycling of crop plants, and building water treatment plants to remove pollutants and clean wastewater.

## 1 Introduction

Agriculture is the backbone source of the human food supply that aims provision of safe products with the least environmental impacts. Food obligation is attained by increasing the crop yield and is achieved by the use of certain agrochemicals (Rafique et al., 2016). Moreover, agriculture is principally based upon two natural resources i.e. soil and water, and dearth in case poses a potential threat to the production of growing food crops (Perveen et al., 2010). Like other developing countries in the world, Pakistan is also a developing agro-based country and is facing freshwater scarcity. To meet water requirements, farmers have a general practice to use sewage and industrial wastewater as irrigation sources which include different heavy metal species including Pb, Zn, Cd, Ni, Fe, Mn, Cr, etc. (Ali et al., 2014a; Ali et al., 2014b; Kanwal et al., 2020; Aslam et al., 2021; Rabiya et al., 2022). Different agrochemicals like synthetic fertilizers and pesticides (xenobiotics) also act as potential sources of heavy metals. Thus, the agricultural system of Pakistan is facing immense heavy metal pollution through such unwise agricultural practices. Such a kind of irrigation is radical for soils, plants, and consumers’ health as well. Different studies have addressed this issue in various cities of Pakistan i.e. in Lahore, Faisalabad, Attock (Rashid et al., 2018), Sargodha (Ahmad et al., 2019), in Karachi (Shehnaz et al., 2019; Aziz et al., 2021) and Sawabi (Ali et al., 2021). On the other, studies have revealed that only 2% of cities are availing wastewater treatment systems in Pakistan, whilst 80% of the cities use untreated discharge for irrigation purposes (Ullah et al., 2018; Shahid et al., 2020; Bashir et al., 2021). Estimates have revealed that >960 thousand million gallons of municipal (> 675) and industrial (>290) wastewater is generated annually (WB-SCEA, 2006), and is adding heavy metals into the intact environment (Sial et al., 2006; Riaz et al., 2020). Among other pollutants, heavy metals are the foremost contributing pollutants due to their potential in changing the physical and chemical characteristics of the ecosystem (Ali et al., 2018; Sobhanardakani, 2019).

Heavy metals are non-essential and non-biodegradable toxic substances with a density greater than 5 g/cm^3^ which is five times more than the density of water (Dai et al., 2006). They are very toxic even used in low concentrations, are deadly unsafe for human health, and produce oxidative stress after entry through the food chain either through natural or anthropogenic source. Moreover, heavy metals also cause liver cirrhosis, renal dysfunction, mental retardation, CNS breakdown, cardiovascular disorder, infertility, and degeneration of the basal ganglia of the brain and ultimately cause death (Sobhanardakani, 2017; Oyugi et al., 2021; Paithankar et al., 2021). For instance, As even at its minute exposure to humans acts as carcinogenic, destroys blood composition, causes anorexia, hair loss, kidney and liver damage, herpes, muscle spasms etc. (Tasleem Jan et al., 2015; Sobhanardakani, 2018; Sobhanardakani, 2018b). Cd is highly toxic element to the human body and specifically targets the vital organs including kidneys and liver, and reduces enzymatic activity related to kidney function (Wuana and Okieimen, 2011; Özcan and Al-Juhaimi, 2012). Chromium is another chemical element and is graded as human carcinogen (Sobhanardakani and Kianpour, 2016). Lead is considered to be the most absorbed environmental pollutant into the human body daily by 80% - 90% *via* consumption of different food items, and affects mental retardation in young children (Krejpcio et al., 2005; Liu et al., 2010). Nickel is an essential element for human body, but its above permissible concentration affects human body at large i.e. Ni associates with the hematotoxicity, genotoxicity, immune-toxicity and carcinogenicity (Akar et al., 2019; Sobhanardakani, 2019).

Focusing on the consequences of the issue, the present study was designed to evaluate the status of heavy metal species in the wastewater of the city Manka sewage canal and sewage wastewater of industrial sites. Likewise, different samples of irrigated soil, and vegetables and crop plants were also collected from different irrigated sites of the study area for metal analysis. Moreover, in present study potential health risk coupled with dietary use of these toxic metal species has been assessed by manipulating the Health Risk Index (HRI).

## 2 Materials and methods

### 2.1 The geographical position of the study area

**Figure 1:**
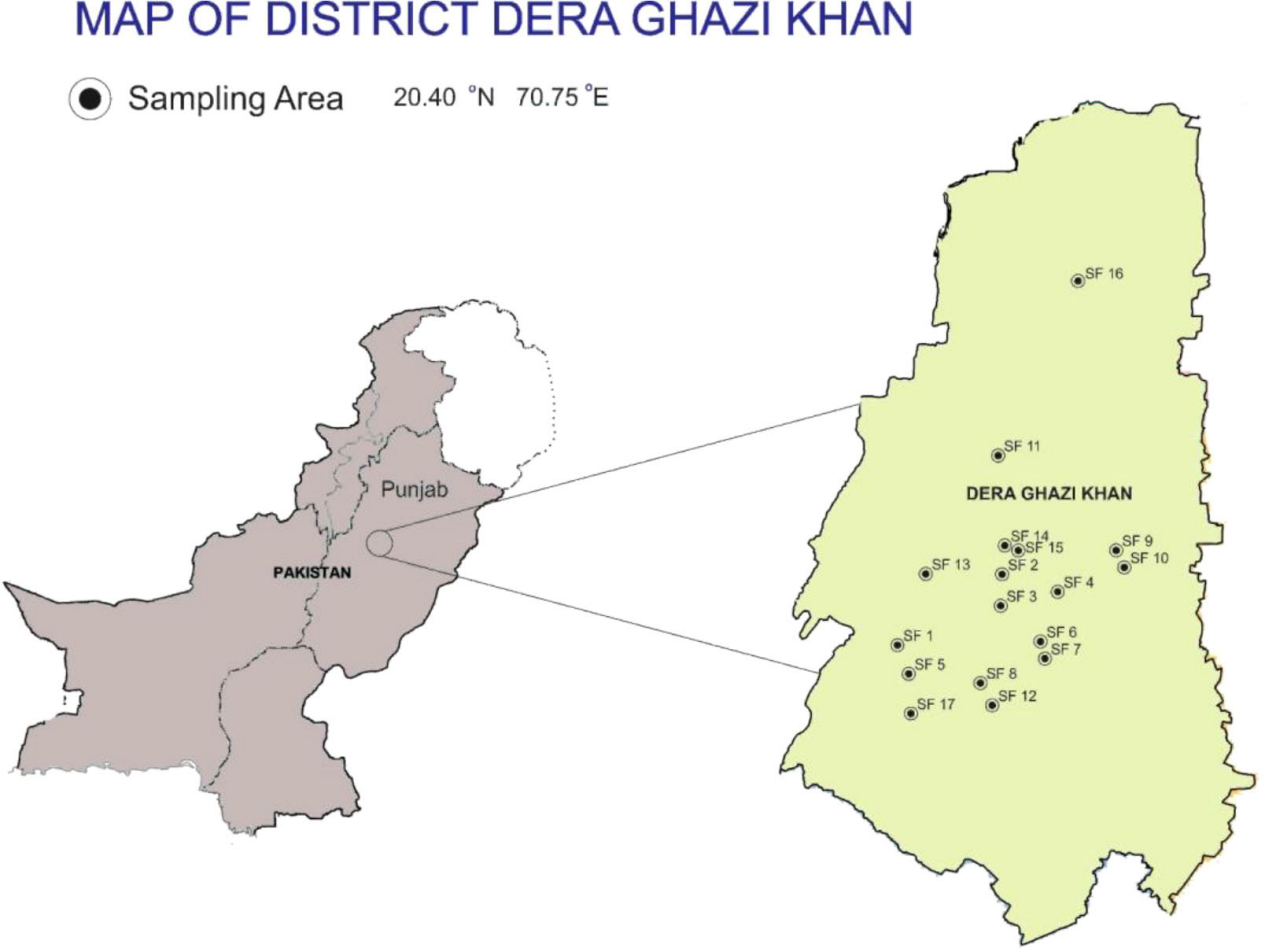
Map of Dera Ghazi Khan District showing sampling sites.

Dera Ghazi Khan is the district of the Province of Punjab (Pakistan), and is located between the river Indus and the Koh-e-Suleiman range. The district coordinates between 20.40 ˚N and 70.75 ˚E (**Figure 1**). This region has 4.1 million hectares of land, out of which, 2.4 million hectares of land is under agricultural use. The district also comprises 68.03-hectare forest land while the rest of the land is either not under cultivation or in social-waste. The study area is almost characterized by a dry atmosphere and very low rainfall following a frosted winter and extremely hot summer. Meteorological data reveals per annum insufficient precipitation in the locale (Latif et al., 2018).

### 2.2 Survey, collection of samples, and Interviews

A total of 17 field sites (SF1-SF17) were selected that are receiving Manka municipal sewage and industrial effluent (textile and pesticide plant) mixed sewage wastewater. For this purpose, a survey was conducted in approximately a 55 km area, during the year 2018-20. The sampling sites were the textile mill (SF1: Paigah sewage drain; 29.57°N, 70.38°E), Chah Poongar wala (SF2: 30.04°N, 70.65°E), Methu wala (SF3: Samina chowk; 30°N, 70.6°E), Chah Somro wala (SF4: Samina chowk; 30.1°N, 70.62°E), Paigah sewage drain (SF5: 29.65°N, 70.44°E), Chah Sinawa wala (SF5: Samina chowk; 30.1°N; 70.62°E), Lohar wala-1 (SF6: 29.96°N, 70.6°E), Lohara wala-2 (SF7: Khoh Nawa; 30°N, 70.54°E), Basti Saddu (SF8: Choti Zerin; 29.84°N, 70.5°E), Gaddai-1 (SF9: 30.02°N, 70.26°E), Gaddi-2 (SF10: 30.02°N, 70.26°E), Yaroo Khosa (SF11: 30.16°N, 70.37°E), sewage drain connecting the outlet of pesticide plant Choti (SF12: 30°N, 70.06°E), Basti Leghari (SF13: 30.03°N, 70.66°E), Manka canal city-1 & 2 (SF14, 15: 30.06°N, 70.63°E), Shah Sadr Din (SF16: 30.27°N, 70.72°E), and Mamoory (SF17: 29.54°N, 70.33), respectively. From either site, three appropriate samples (*n* = 3 × 17 = 51) of either wastewater, soil (top to 30 cm deep soil), and plants were collected, aggregated, and preserved in identifiable sealed packs. These samples were prepared for the subsequent metal analysis. During the survey and sample collection, farmers (120 respondents) were also asked about necessary information regarding awareness, use, hazards of wastewater and diet requirement (mg person ^-1^ day^-1^), body weight, etc. through a questionnaire.

### 2.3 Spectroscopic analysis of samples

Wastewater samples were processed to determine pH and EC (µS/cm) following the method Jackson (1967). Total dissolved solids (TDS) of wastewater were assessed by an evaporating method at 300 ˚C using a hot plate. Heavy metal analysis of samples was performed by using an atomic absorption spectrometer (AAS: Perkin Elmer) following Welz and Sperling (1999).

#### 2.3.1 Metal detection in wastewater

A 10 ml of filtered wastewater sample was used for acid digestion. For this purpose, 2 ml of HNO_3_ and 5 ml of HCl were added to the sample wastewater and heated at 95°C till the appearance of a colorless solution phase. After cooling the solution, the volume of the sample was raised to 50 ml by using distilled water. A series of standard solutions of selected metals were run to assess the unknown values of heavy metals in water samples. Different metals were analyzed at the appropriate wavelength (Al 309 nm, As 193.7 nm, Cd 248.3 nm, Cr 357.9 nm, Cu 324.8 nm, Fe 309 nm, Mn 279.5 nm, Pb 283.3 nm, Zn 228.8 nm, and Ni 232 nm).

#### 2.3.2 Metal detection in soil and vegetable/crop samples

The detection of metals in the collected soil and plant samples was tested by the hot acid digestion method. One g dried soil/plant sample was digested in 15 ml of the acid mixture in a 5:1:1 ratio (70% HNO_3_, 70% H_2_SO_4_, and 65% HClO_4_). After cooling, a colorless acidic aliquot was filtered (by Whatman No. 42) and diluted with distilled water. Analysis was carried out for different metals at analytical spectral lines i.e. Al 309 nm, As 193.7 nm, Cd 248.3 nm, Cr 357.9 nm, Cu 324.8 nm, Ni 232 nm, Fe 309 nm, Mn 279.5 nm, Pb 283.3 nm, and Zn 228.8 nm.

#### 2.3.3 Quality control analysis and assurance

For metal analysis, chemicals of analytical grade were used from Merck-Germany. AAS was calibrated with the series of standard solutions from the metal stock-solution using calibration curve. Double deionized water was used to prepare the stock-solution. Likewise, high quality glass ware (Merck-Germany) was used after cleaning with diluted HNO_3_ to avoid the possible contamination. For more accuracy of the results, analysis was repeated thrice for each sample following the standard reference procedure.

#### 2.3.4 Metal frequency

Metal frequency was calculated for different metals in the selected irrigated sites using the following formula:

F (%) = No. of samples with a particular metal/total No. of samples analyzed

#### 2.3.5 Metal accumulation factor

Transfer of metal content from soil to the growing plants was estimated by the method of Raskin et al. (1994) using the following formula:


ACF= PU/MT

Where, PU = metal content in whole plant (mg kg^-1^), MT = total metal content in soil (mg kg^-1^).

### 2.4 Estimation of daily metal intake

Daily intake of vegetables/edible parts of crops in adults (mg person^-1^ day^-1^) was calculated by data obtained during the survey/interview of 120 male farmers through a questionnaire. The following equation was used to calculate DMI value (Chary et al., 2008):


$$DMI\;=\;C_{m}\times \;C_{f}\times \;D_{daily\;food\;intake/}B_{bw}$$

where, C*_m_*, C*_f_*, D _daily food intake_ and B*_bw_* are metal content in collected plants (mg kg^-1^), conversion factor (0.085), daily ingestion of contaminated vegetables/crops, and average body weight, respectively. The respondents have an average 50 kg body weight, and were inquired about their daily food intake of vegetables or edible parts of crops (wheat bread + corn grains) from the sampling sites (was found 0.600 kg day^-1^ approximately).

### 2.5 Estimation of health risk index

Probable health risks due to the daily intake of metals through an approximate consumption of contaminated food at sampling sites were calculated as HRI. The health risk index (HRI) was determined based on the relation of DMI to oral reference dose (Rfd). HRI was calculated as in the following equation (Stephens et al., 2001):


HRI = DMI/Rfd

Where, DIM is the daily intake of metals and Rfd represents a reference oral dose. Rfd value for Cd, Cr, Cu, Fe, Ni, Mn, Pb and Zn is 0.001, 1.5, 0.04, 0.7, 0.02, 0.014, 0.004, 0.3 (mg/kg bw/day), respectively (US-EPA IRIS, 2006). Moreover, HRI value >1 for the possible consumption of plant species was used as unsafe for the consumers (US-EPA, 2002).

### 2.6 Statistical analysis

Statistical analysis of data was performed by ANOVA and standard deviation test using SPSS statistical package (V. 20) while correlation and variance of metals in different samples of the study area were performed by principal component analysis (PCA) using MVSP (V. 3.2).

## 3 Results

### 3.1 Characterization of wastewater and soil samples

**Table 1** presented different characteristics of wastewater being used as an irrigation source at 17 agricultural sites of the District Dera Ghazi Khan. Data from mean values significantly showed pH and TDS within the control limits while EC was found above the permissible limit. Results of these parameters pertaining to specific irrigated soils (SF1-SF17) showed a marginal pH value in SF3, SF5, SF6, and SF17, while SF16 and SF17 (Manka canal city area) showed high pH (8.8-8.9) than the normal values of pH (6.5-8.4). Likewise, the TDS of water in all sites was below the safe limit (2000 ppm), but in the city, area TDS was quite marginal (1902 ppm). The mean value of EC showed to be elevated for 58.8% of samples (above 3000 µS/cm), while 29.4% of samples showed a safe but increasing trend in EC (2650-2749) and 11.8% were within the safe limit (2394-2470). The highest value of EC was recorded for SF5 (3750: Paiga sewage drain) and SF15, 16 (3289-3471: Manka canal city area).

**Table 1:** (A) Characteristics of wastewater and heavy metal availability (mg L^-1^) in wastewater used for irrigation purpose.

Parameter	Range	Mean value	± SD	*p*	Frequency (%)	Permissible limit	Reference (s)
pH	6.7 - 8.8	7.73	0.71	–	100	6.5 - 8.4	FAO (1985); Pescod (1994)
	1270 - 1902	1526.4	168	–	100	2000	
TDS (ppm)							
EC (µS/cm):	2470 - 3750	3003	373.8	–	100	< 3000	
Al	0.1 - 0.66	0.24	0.19	^***^	76.5	1.5	WHO/FAO (2001), FAO (1985)
As	0.01 - 0.2	0.09	0.06	^***^	64.7	0.1	Ayers and Westcot (1985)
Cd	0.004 - 0.13	0.04	0.01	^*^	82.3	0.01	
Cr	0.06 - 2.92	0.4	0.71	^***^	100	0.1	
Cu	0.02 - 0.24	0.09	0.07	^*^	88.2	0.2	
Fe	0.005 - 0.16	0.09	0.06	^***^	58.8	5	
Mn	0.01 - 1.0	0.26	0.29	^***^	88.2	0.2	
Ni	0.02- 0.19	0.08	0.08	^***^	76.5	1	
Pb	0.01 - 1.17	0.19	0.33	^***^	100	2	
Zn	0.01 - 1.95	0.4	0.52	^***^	88.2	2	
**(B): Heavy metal availability in soil (mg kg^-1^) irrigated with wastewater**							
Al	0.04 - 1.41	0.49	0.4	^***^	88.2	–	WHO/FAO (2001), Ministry of Environment Finland (2007)
As	0.001 - 0.13	0.06	0.04	ns	58.8	5	
Cd	0.07 - 0.43	0.17	0.11	ns	82.3	1	
Cr	2.01 - 13.5	11.32	3.42	^***^	100	100	
Cu	0.11 - 2.08	0.67	0.56	^***^	82.3	100	
Fe	1.22 - 4.0	2.13	3.02	^***^	82.3	10	
Mn	0.23 - 3.19	1.2	0.97	^***^	76.5	–	
Ni	0.87 - 7.87	3.71	2.23	^***^	88.2	50	
Pb	0.13 - 2.08	1.05	0.64	^***^	100	60	
Zn	1.05 - 7.3	3.33	2.17	^***^	88.2	200	

p, Significant level ^***^ at 0.1, ^*^ at 0.05); ns, non-significant; TDS, Total Dissolved Solids; EC = electrical conductivity.

Analysis revealed presence of different heave metals (Al, As, Cd, Cr, Cu, Fe, Mn, Ni, Pb, Zn) in the irrigated wastewater from selected sampling stands. For these stands, frequency (% age) of trace metals was varying, and however, was 100% in the case of Cr and Pb as found in all the stands. Likewise, heavy metal concentration in irrigating water at the sampling sites was variable (**Table 1A**). The content of As, Cd, and Cr was above the acceptable limit (SF1, SF5, SF8, SF9, SF11, SF13, SF14, SF16), while Cu, Pb, and Mn showed a marginal overlook. Chromium was 2.92 mg. L^-1^ in textile mill sewage drain (SF1) followed by 1.19 mg/L in water receiving outlet drain of pesticide plant (SF13). Moreover, the city Manka canal also showed a marginal level of Cr (0.18-0.21 mg L^-1^). Similar findings were about Cd accumulation. Sampling stands SF1, SF12, and 13 showed a marginal concentration of Cu as compared to the safe limit, while Pb concentration was high (1.03-1.17 mg L^-1^) but below the safe limit. Heavy metal content in soil varied among the sites (SF1-SF17). Among the detected metals, Cr and Pb were the most frequent. Areas irrigated with the city Manka canal i.e. SF1, SF 7-8, SF 10-11, SF5, SF13, and SF14 were noticed with high amounts of metals (Cr, Cu, Ni, and Fe) but below the permissible limit. However, the concentration of heavy metals recorded in irrigated soils of the study area was found safe and below the permissible limit (**Table 1B**).

### 3.2 Concentration of heavy metals in vegetables and crop plants

**Table 2** comprises different concentration levels of heavy metals in vegetables and crop plants that were grown up on municipal and industrial mixed sewage wastewater along with the permissible limits set by WHO/FAO (Aderinola et al., 2012). Results obtained from different growing sites have significantly shown eminent metal content in collected vegetables and crop plants. Among the metals, concentration of Mn (2.31-18.71 mg/kg), Ni (0.72-1.53 mg/kg) and Cr (2.4-4.61 mg/kg) were found above the acceptable limit while Pb (0.004-0.34 mg/kg) showed to be marginal in few cases. In overall, regarding metal concentration, a descending pattern of metals was observed as: Fe (12.7-329.8 mg/kg) > Cu (7.19-33.5 mg/kg) > Zn (8.72-29.3 mg/kg) > Mn (2.31-18.71 mg/kg) > Cr (1.22-4.61 mg/kg) > Ni (0.67-1.53 mg/kg) > Pb (0.004-0.34 mg/kg) > Cd (0.11-0.18 mg/kg) > As (0.003-0.001 mg/kg). All plant samples showed an exceeding concentration of Mn. Chromium was also found exceeding in the plant samples except for luffa and white corn. Likewise, Ni was abundant in plant samples except for luffa, gourd, and cabbage. Cadmium was observed to be marginal and within the safe limit in brinjal, wheat, and spinach only. Exclusively, brinjal, wheat, tomato, and spinach were the potential species that showed a tendency of almost higher metal concentration.

**Table 2:** Concentration of heavy metal content (mg kg^-1^) in vegetables/crop plants irrigated with wastewater.

Vegetable/Crop	part used	As	Cd	Cr	Cu	Fe	Mn	Ni	Pb	Zn
Brinjal (*Solanum melongena* L.)	Fruit	0.001	0.18	4.61	21.5	252.1	11.3	1.08	0.33	23.4
Red Corn (*Zea mays* L.)	Shoot	0	0.12	2.4	20	58.3	5.9	1	0.34	16.1
White Corn (*Zea mays* L.)	Shoot	0	0	1.34	13.8	64.6	3.4	0.67	0.22	17.5
Wheat (*Triticum vulgare* L.)	Grain	0.0002	0.14	2.61	22.4	103	3.8	2	0.17	29.3
Tomato (*Lycopersicum esculentun* L.)	Fruit	0.0002	0.12	3.02	10.6	69.4	4.6	1.23	0.15	26
Luffa (*Luffa cylindrical* L.)	Fruit	0	0	1.22	12.8	12.7	2.3	0	0.004	11.3
Apple Gourd *(Praecitrullus fistulosus* L.)	Fruit	0	0.11	3.7	14.2	14.21	3.1	0	0.23	19.8
Cabbage (*Brassica oleracea* L.)	Flower	0	0.13	3	24.1	29.5	6.1	0	0.09	0
Spinach (*Spinacia oleracea* L.)	Laves	0	0.14	4.17	33.5	329.8	18.8	1.53	0.09	14.2
± SD		0.0003	0.063	1.16	7.19	111.63	5.3	0.72	0.11	8.72
*P*		**^ns^**	^**^	^***^	^***^	^***^	^***^	^***^	^***^	^***^
WHO/FAO permissible limit (Aderinola et al., 2012)		0.5	0.2	2.3	73	425	0.2	0.1	0.3	99

p, Significant level: *** at 0.1, ** at 0.01, ns, non-significant.

### 3.3 Metal accumulation factor of metals from soil to plants

Metal accumulation factor is the potential of a plant to absorb metal content from the intact metal-enriched soil. Different plants show varying potential toward metal contents in this regard. **Table 3** summarizes the metal accumulation/transfer factor in plants collected from the study area. Mean ACF value in these plants was 48.7 (Fe) > 28.7 (Cu) > 5.5 (Mn) > 5.26 (Zn) > 0.62 (Cd) > 0.26 (Cr) > 0.22 (Ni) > 0.17 (Pb) > 0.0026 (As). Overall, brinjal, red corn, wheat, tomato, and spinach showed higher values of ACF than other plant species.

**Table 3:** Accumulation factor of metal content (ACF) from soil to plant.

Vegetable/crop	As	Cd	Cr	Cu	Fe	Mn	Ni	Pb	Zn
Brinjal	0.02	1.1	0.41	32	118.4	9.4	0.29	0.31	7.0
RED Corn	0	0.7	0.21	30	27.4	5	0.27	0.32	4.8
White Corn	0	0	0.12	21	30.3	2.9	0.18	0.21	5.2
Wheat	0.0033	0.8	0.23	33.4	48.4	3.1	0.54	0.16	8.8
Tomato	0.0033	0.7	0.27	16	32.6	3.8	0.33	0.14	7.8
Luffa	0	0	0.11	19.1	5.96	1.9	0	0.004	3.4
Apple Gourd	0	0.6	0.33	21.2	6.7	2.6	0	0.22	5.9
Cabbage	0	0.8	0.26	36	13.8	5.1	0	0.08	0
Spinach	0	0.8	0.37	50	154.8	15.6	0.41	0.09	4.3
Mean ACF	0.003	0.62	0.26	29	48.7	5.5	0.22	0.17	5.3

### 3.4 Daily metal intake and health risk index of heavy metals

DIM values calculated for adults are presented in **Table 4**. Obtained data revealed that daily ingestion of metals was high for brinjal, wheat, tomato, cabbage, and spinach as compared to corn, luffa, and gourd vegetables grown on wastewater. The trend of DIM was found to be the highest for Fe (0.105809) > Cu (0.019595) > Zn (0.017862) > Mn (0.006727) > Cr (0.002955) > Ni (0.000851) > Pb (0.000184) > Cd (0.0001071) > As (5.1E-7). The health risk index for heavy metals by eating contaminated vegetables and crop plants was calculated from DMI values (**Table 5**). The maximum HRI was found for spinach (6.4) > wheat (6.4) > brinjal (5.9) > tomato (4.7) > red corn (4.5) > apple gourd (4.3) > white corn (3.8) > cabbage (3.1) > luffa (2.9). Likewise, the order of HRI from high to low risk value was found as Cu (19.6) > Zn (17.9) > Cr (2.95) > Ni (0.85) > Mn (0.48) > Fe (0.15) > Cd (0.11) > Pb (0.05) > As (3.2E-6). HRI value showed spinach to be the most health-risk leafy vegetable grown in wastewater of the area, while luffa represented to have the least health-risk possibility. On the other hand, Cu, Zn and Cr metals showed the highest HRI value in wastewater-irrigated plants.

**Table 4:** Estimation of daily metal intake (mg person^-1^ day^-1^) through consumption of vegetable/crop food.

Vegetable/crop	As	Cd	Cr	Cu	Fe	Mn	Ni	Pb	Zn
Brinjal	0.000000102	0.0002	0.005	0.02	0.26	0.01	0.001	0.0003	0.02
RED Corn	0	0.0001	0.002	0.02	0.06	0.01	0.001	0.0003	0.02
White Corn	0	0	0.0014	0.01	0.07	0.003	0.001	0.0002	0.02
Wheat	0.000000204	0.0001	0.003	0.02	0.10	0.004	0.002	0.0002	0.03
Tomato	0.000000204	0.0001	0.003	0.01	0.07	0.005	0.001	0.0001	0.03
Luffa	0	0	0.002	0.01	0.01	0.002	0	4.1E-06	0.01
Apple Gourd	0	0.0001	0.004	0.01	0.01	0.003	0	0.0002	0.02
Cabbage	0	0.0001	0.003	0.02	0.03	0.01	0	9.2E-05	0
Spinach	0	0.0001	0.004	0.03	0.34	0.091	0.002	9.2E-05	0.01
MEAN DMI	5.1E-7	0.0001	0.003	0.02	0.11	0.07	0.001	0.01	0.02

**Table 5:** Assessment of health risk index (HRI) of metals upon consumption of contaminated vegetable/crop food.

Vegetable/crop	As	Cd	Cr	Cu	Fe	Mn	Ni	Pb	Zn
Brinjal	0.000001	0.20	4.7	21.9	0.37	0.82	1.10	0.08	23.9
RED Corn	0	0.13	2.4	20.4	0.85	0.43	1.02	0.09	16.4
White Corn	0	0	1.4	14.1	0.94	0.25	0.68	0.06	17.8
Wheat	0.000002	0.14	2.7	22.8	0.15	0.27	2.04	0.04	29.9
Tomato	0.000002	0.12	3.1	10.8	0.10	0.33	1.25	0.04	26.5
Luffa	0	0	1.2	13.1	0.18	0.17	0	0.001	11.6
Apple Gourd	0	0.11	3.8	14.5	0.21	0.22	0	0.06	20.2
Cabbage	0	0.13	3.1	24.6	0.43	0.44	0	0.023	0
Spinach	0	0.14	4.2	34.2	0.48	1.40	1.60	0.023	14.5
**Mean HRI-value**	**3.2E-6**	**0.11**	**2.9**	**19.6**	**0.15**	**0.48**	**0.85**	**0.05**	**17.9**

### 3.5 Principal component analysis of metals

**Figure 2:**
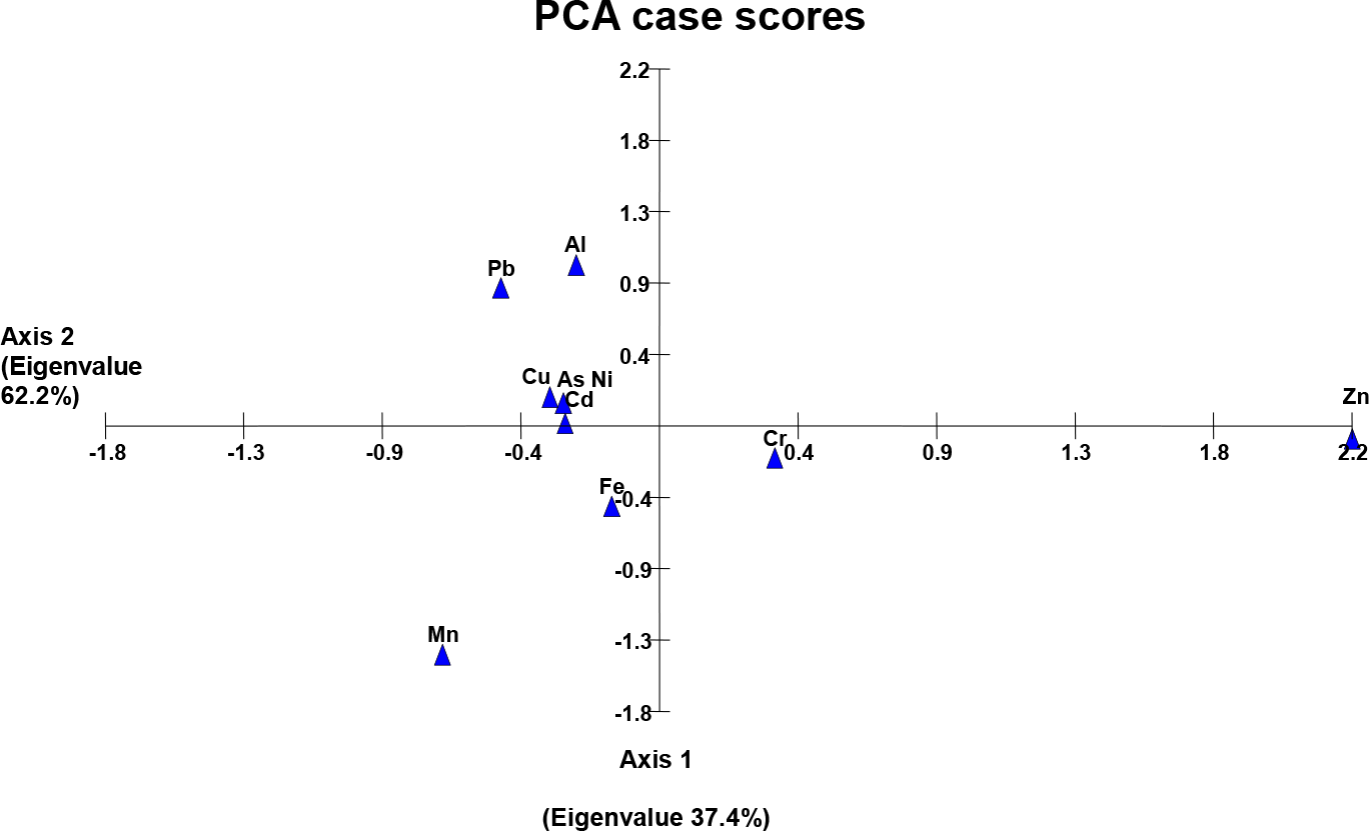
Heavy metals in wastewater used for irrigation.

**Figure 3:**
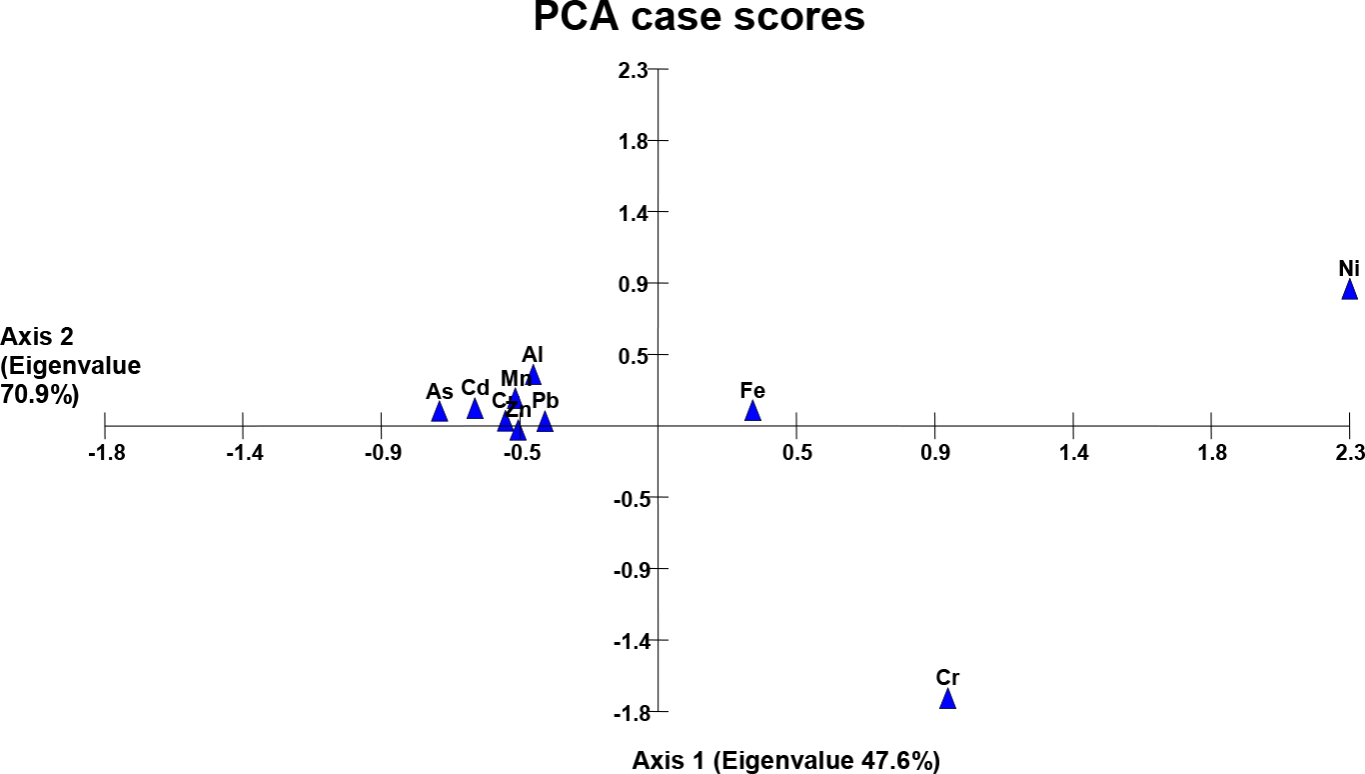
Heavy metals in soil irrigated with wastewater.

Comparison of two axes of the PCA bi-plot showed a strong correlation among the detected heavy metals (axis-2 labeled with high eigen-value than axis-1) except a significant variance along axis-1 in the case of Cr and Zn (wastewater), Cr, Ni, and Fe (soil samples) while the variance in the case of Cu, Zn and Fe was observed in plant samples. This kind of metal distribution explained the dimensional correlation/variance among the heavy metals in the study area (**Figures 1**– **3**).

## 4 Discussion

During the survey and sample collection, it has been observed that the farmers (respondents) of Dera Ghazi Khan District are using municipal and industrial-mixed sewage wastewater as a source of irrigation for a long. The farmers were also assessed about their knowledge of wastewater and HMs hazard through a questionnaire and were found unaware or have a bit of knowledge about the hazard to growing plants, irrigated soil, and consumers. During the present study, the heavy metal availability has also been confirmed in the collected samples of wastewater, soils, and even in the growing plants from different irrigated sites (SF1-SF17). The subsequent analyses have pretentiously confirmed the presence of different heavy metals in these collected samples, and have predicted the potential health hazards to the subsequent consumers of the area. Moreover, a mean value of pH of the wastewater has been observed within the safe limit except in a few sites following an increasing trend of TDS but within the safe limit. As concerns EC of the wastewater, the mean value was found to be exceeding the safe limit, and individually most of the sites (SF-1, 3, 5, 15, 16, 17) have an exceptional value of EC. From **Table 1**, it is observed that in the wastewater upper range of trace elements like Cr, Cd, and Mn have exceeded the permissible limits, whilst Al, Cu, Fe, Pb, Zn, and Ni were observed within the safe limit. However, arsenic (As) was observed at the margin in some sites (SF-9, 13). These metals need careful management and monitoring from exceeding in the future. Hence, exceeding values of pH, TDS, EC and different heavy metals in the wastewater have turned it to be unfit for irrigation purposes in the study area. The findings of our study are supported by Kumar et al. (2018) that sewage and industrial wastewater were found to be unfit for irrigation purposes due to exceeding values of pH, TDS, and EC that changed the physic-chemical properties of irrigated soils and plants (spinach, fenugreek, and coriander). The wastewater was also found with a high amount of heavy metals, and the same was detected in the grown vegetables in an order of Cd > Zn > Fe > Mn > Pb > Cr > Cu. Likewise, the findings of Ishaq et al. (2020) showed the above permissible limits of pH, TDS, and EC in sewage wastewater of Peshawar city along with the varying concentration of heavy metals i.e. Cd, Pb, Cu, Ni, Zn, and Fe. These heavy metal traces were also observed in tomato plants irrigated with such metal-enriched sewage wastewater. According to a study by Hassan et al. (2022), the textile wastewater was also found with differentiated physiological parameters (EC, TDS) and heavy metals (Cd, Ni, Cr, Cu, Zn, Fe, Pb) that were exceeding the recommended limits. A high pH of wastewater increases the pH and alkalinity of irrigated soil which potentially tends to the availability of heavy metals in the soil and contaminate the food chain and vice versa. Such an affected food chain poses health risks to subsequent consumers (Ali et al., 2015a; Mwamba et al., 2020; Waheed et al., 2020).

Results about metals in the soils (**Table 1B**) irrigated with the wastewater have revealed the metal accumulation which was below the permissible limit; however, Cr and Pb were the most frequent (100%) among the metals. Likewise, the mean accumulation of heavy metals for all 17 stands has also revealed an elevated concentration of chromium (11.32 mg/kg). Foregoing deep insight, by differentiating the irrigated sites, ten sites SF-5, SF 7-10, and SF 12-17 have been found with the elevated metal contents (Cr, Cu, Ni, Pb, and Zn) under the cultivation of the cotton crop, might be due to excessive use of fertilizers and pesticides along with the wastewater application as reported earlier (Rafique et al., 2016; Gill et al., 2016; Gill et al., 2017). Briefly, available data from our analyses have revealed that sewage and industrial wastewater is unfit for irrigation purpose and has potential hazards due to the accumulation of metals. Earlier studies by Rafique et al. (2016) and Latif et al. (2018) also supported the findings of the present investigation and confirmed the availability of different heavy metals in cultivated soil of various areas of the Dera Ghazi Khan district. These areas were irrigated with sewage and industrial wastewater for a long time. Such a kind of irrigation was the potential source of heavy metal accumulation in agro-soils and grown vegetables. Heavy metals (mg/kg) were detected in the irrigated soils and vegetables were in the order of Cr [(17.89) > Ni (9.24) > Zn (2.31) > Pb (0.84) > Cu (0.29) > Cd (0.186); and Mn (137.3) > Cr (6.62) > Fe (968)]. Chromium, Mn, Fe, Ni, and Pb were exceeding the FAO/WHO limits (Aderinola et al, 2012; Rafique et al., 2016; Latif et al., 2018; Guo et al., 2021).

In the present study, besides heavy metal accumulation in the agro-soil system through wastewater, the collected samples of vegetables and crop plants have been revealed to be metal-enriched. It has shown an effective metal transfer from contaminated soil to the grown plants with the exceeding limits of Cr, Ni, Mn, and Pb. However, As, Cd, Cu, and Zn traces have been found within the safe limit (**Table 2**). Interpretation of HRI has revealed the possible potential risk of heavy metal contaminated plants in the order of spinach > wheat > brinjal > tomato > red corn > apple gourd > white corn > cabbage > luffa. The health risk assessment of different metals has been found as Cu > Zn > Cr > Ni > Mn > Fe > Cd > Pb > As (**Table 5**). These toxic metals put serious health risks to subsequent consumers interacting through the food chain (Ali et al., 2015b; Ali et al., 2015c; Dziwornua et al., 2018; Zuo et al., 2019; Tian et al., 2021) cause damage to DNA and proteins, and become carcinogenic in humans (Edelstein and Ben-Hur, 2018; Witkowska et al., 2021). Exposure to heavy metals may also result in skin-lung disorder, epigastric pain, hemorrhage, etc. (Das and Roychoudhury, 2014). Cattle milk is used as a nutritive source of food for all age groups including infants. But it possesses serious health risks due to heavy metals in the milk transferred from cattle after consumption of metal-contaminated fodder crops previously irrigated with sewage water, synthetic fertilizers, fungicides, pesticides, and industrial wastewater (Shar et al., 2021). In agreement with these earlier studies, our findings regarding the detection of different heavy metals in different vegetables and crop plants irrigated with metal-enriched wastewater have predicted potential health risks to the consumers of the study area through calculation of HRI (**Table 5**).

**Figure 4:**
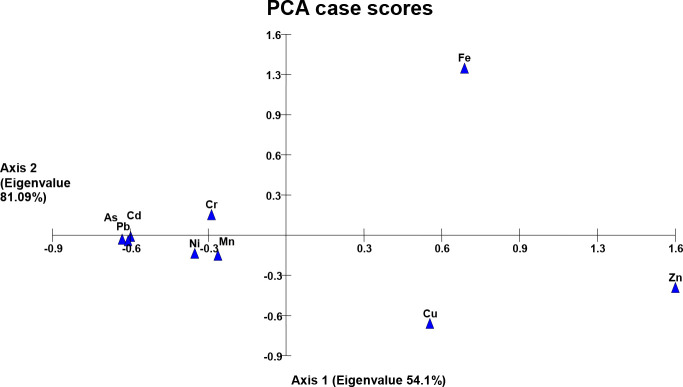
Heavy metals in vegetables and crop plants irrigated with wastewater.

Results of PCA have shown a distribution pattern of heavy metals in the agricultural system of the study area, and a pronounced variation along axis 1 following a strong correlation among the metals along axis 2. **Figure 2** shows a strong variance in Cr and Zn in the wastewater collected from different sites (axis 1). The reason may be the exceptional concentration of these metals in sewage wastewater possibly receiving the metal residues from the textile mill (SF1, SF5) and pesticide plant wastewater (SF 13, SF 9). Heavy metals in soil samples irrigated with the wastewater have also shown a correlation of metals along axis 2, while Fe, Ni, and Cr are the exception on axis 1. We have observed the maximum concentration of these three metals in almost all the collected soil samples as compared to the concentration found in wastewater samples (**Figure 3** and **Tables 1A, B**). This kind of metal accumulation in the soil samples may be due to the excessive use of pesticides or synthetic chemical fertilizers besides using municipal or industrial mixed sewage wastewater as a possible source of heavy metals (Rafique et al., 2016; Ulhassan et al., 2019). Likewise, subsequent plant samples have also shown a strong variance regarding Cu, Zn, and Fe metals (**Figure 4**). This may be due to the specific mineral composition of collected plants (as in the case of spinach having more Fe-content), or excessive application of pesticides and synthetic fertilizers specifically absorbed by some growing plants.

Environmental pollution brought by addition of heavy metals in the agricultural system has been found as major health risk for humans and the environment. Due to their nature of toxicity and non-biodegradable property, these heavy metals remain present in the environment. Removal of such toxic entities is being made by imposing bioremediation technique - an effective tool to clean the environment. Microorganisms (aerobes, anaerobes) are used to treat contaminated soils for effective elimination, degradation and immobilization of heavy metals (Bala et al., 2022). Moreover, the source water pollution can be focused on remediation of wastewater and organic pollutants using some membrane or anaerobic microbial bioreactors (Ahmad et al., 2020; Naz et al., 2021).

## 5 Conclusion

The present study has revealed that wastewater samples collected from different sites of the study area have varying physico-chemical properties and are loaded with heavy metal trace elements. The concentration of As, Cd, and Cr was exceeding the acceptable limit, while Cu, Pb, and Mn showed to be marginal. Unremitting irrigation with wastewater has caused an accumulation of toxic metals in the subsequent agricultural land sites. However, soil samples showed metal accumulation below the permissible limit, and however, Cr and Pb were the most frequent (100%) among the metals. Likewise, the mean accumulation of heavy metals for all 17 stands has also revealed an elevated concentration of chromium (11.32 mg/kg) followed by Cu, Ni, Pb, and Zn. Samples of vegetables and crop plants were also revealed to be metal-enriched with the exceeding contents of Cr, Ni, Mn, and Pb. Based on DMI, HRI revealed the possible potential risk of vegetables and crop plants contaminated with heavy metals in the order of spinach > wheat > brinjal > tomato > red corn > apple gourd > white corn > cabbage > luffa. Likewise, health risk assessment of different metals was observed in the order of Cu > Zn > Cr > Ni > Mn > Fe > Cd > Pb > As. The present study also suggests that prolonged application of wastewater as an irrigation source may cause severe health risks to subsequent consumers. Further, it is also suggested that an imperative notice and awareness to the people is needed for appropriate regulation and monitoring the municipal and industrial effluents as wastewater. Different bioremediation techniques and water treatment plants could be a good tool to revive the contaminated agricultural system of the study area. Moreover, in the future, besides metal pollution the organic pollution and role of microorganisms will be considered. However, it needs attention of the scientific community.

## Data availability statement

The original contributions presented in the study are included in the article/supplementary material. Further inquiries can be directed to the corresponding authors.

## Author contributions

MIA, SZ and HA created the research plan, performed the experiments of MIA Ph. D work, analyzed the data and write down the manuscript. HA, IA, MS, BA worked as technical advisors and helped in the experiments. KN, RI, D-QD, SB, IA, UAH helped in revising, editing and funding acquisition for the manuscript. All authors contributed to the article and approved the submitted version.
